# Heterogeneous Responses to Changes in Inhibitory Synaptic Strength in Networks of Spiking Neurons

**DOI:** 10.3389/fncel.2022.785207

**Published:** 2022-02-24

**Authors:** H. Y. Li, G. M. Cheng, Emily S. C. Ching

**Affiliations:** Institute of Theoretical Physics and Department of Physics, The Chinese University of Hong Kong, Shatin, Hong Kong SAR, China

**Keywords:** neuronal networks, spiking neuron model, bursts, changes in inhibition, heterogeneous responses

## Abstract

How does the dynamics of neurons in a network respond to changes in synaptic weights? Answer to this question would be important for a full understanding of synaptic plasticity. In this article, we report our numerical study of the effects of changes in inhibitory synaptic weights on the spontaneous activity of networks of spiking neurons with conductance-based synapses. Networks with biologically realistic features, which were reconstructed from multi-electrode array recordings taken in a cortical neuronal culture, and their modifications were used in the simulations. The magnitudes of the synaptic weights of all the inhibitory connections are decreased by a uniform amount subjecting to the condition that inhibitory connections would not be turned into excitatory ones. Our simulation results reveal that the responses of the neurons are heterogeneous: while the firing rate of some neurons increases as expected, the firing rate of other neurons decreases or remains unchanged. The same results show that heterogeneous responses also occur for an enhancement of inhibition. This heterogeneity in the responses of neurons to changes in inhibitory synaptic strength suggests that activity-induced modification of synaptic strength does not necessarily generate a positive feedback loop on the dynamics of neurons connected in a network. Our results could be used to understand the effects of bicuculline on spiking and bursting activities of neuronal cultures. Using reconstructed networks with biologically realistic features enables us to identify a long-tailed distribution of average synaptic weights for outgoing links as a crucial feature in giving rise to bursting in neuronal networks and in determining the overall response of the whole network to changes in synaptic strength. For networks whose average synaptic weights for outgoing links have a long-tailed distribution, bursting is observed and the average firing rate of the whole network increases upon inhibition suppression or decreases upon inhibition enhancement. For networks whose average synaptic weights for outgoing links are approximately normally distributed, bursting is not found and the average firing rate of the whole network remains approximately constant upon changes in inhibitory synaptic strength.

## 1. Introduction

Synaptic plasticity, the modification of the strength of synaptic connections in response to activity, has long been proposed to play an important and fundamental role in learning and memory (Hebb, 1949). Extensive studies have demonstrated the various forms and mechanisms of synaptic plasticity in both short-term as well as long-term manners (Brown et al., 1990; Bear and Malenka, 1994; Malenka and Nicoll, 1999; Bi and Poo, 2001; Bi, 2002; Zucker and Regehr, 2002; Citri and Malenka, 2008; Bailey et al., 2015). When synapses are strengthened by activity, the stronger synapses are expected to lead to higher activity and, therefore, it is commonly believed that activity-dependent synaptic plasticity is a positive feedback process that would lead to instability (Abbott and Nelson, 2000) and a number of stabilization mechanisms have been suggested (Chen et al., 2013; Bannon et al., 2020). Using experiments and simulations on a neuron of the lobster and on a model neuron, it was found that the effect of changes in synaptic strength saturates and additional changes beyond the saturation point produce no further changes in the dynamics of the neuron (Prinz et al., 2003). This result thus suggests that changes in the strength of the synapses onto a neuron do not necessarily lead to changes in the spiking activity of that neuron. Moreover, the effect of changes in the strength of synapses on the dynamics of a neuron can be significantly influenced by the connections of this neuron to other neurons in a network. Thus direct studies answering the question of how the dynamics of neurons in a network would be altered by changes of synaptic strength would be important for a full understanding of synaptic plasticity.

Many computational models of networks of excitatory and inhibitory neurons have been used to study different aspects of the brain systems (Einevoll et al., 2019). Different levels of abstraction have been used to model neurons, from Hodgkin-Huxley type models with detailed ionic mechanisms (e.g., Destexhe et al., 1996; Andreev et al., 2019) to simple leaky-and-fire model and phenomenological models with in-between complexity (e.g., Tomov et al., 2014; Zerlaut et al., 2018; Izhikevich and Edelman, 2020; Górski et al., 2021). At the network level, networks with generic properties allow us to gain qualitative insights of the possible wide range of dynamics (Brunel, 2000) while detailed large-scale networks that mimic real brain regions have been constructed (e.g., Traub et al., 2005; Potjans and Diesmann, 2014; Markram et al., 2015; Arkhipov et al., 2018; Schmidt et al., 2018; Izhikevich and Edelman, 2020) to aim for a full understanding of the brain.

In this article, we report our numerical study of how the dynamics of neurons in a network is altered upon a suppression of the inhibition. We performed simulations of a model of stochastic spiking neurons connected by conductance-based synapses and studied how the spontaneous activity of the neurons would change when the magnitudes of all the inhibitory synaptic weights are decreased. We used networks of biologically realistic features, which were reconstructed from multi-electrode array recordings taken in a cortical neuronal culture, and their modifications in the simulations. Our simulations reveal the surprising result that the responses of neurons are heterogenous and the firing rate does not increase for all the neurons. While some neurons exhibit an expected increase in the firing rate, other neurons exhibit a decrease or no change in the firing rate. In comparison with networks with an applied suppression of inhibition, the original networks can be viewed as networks with an applied enhancement of suppression. Hence, our results imply that heterogeneous responses also occur for an enhancement of inhibition. In addition, we have studied the effects of network architecture and our results demonstrate that the distribution of average synaptic weights of the outgoing links of the network plays a crucial role in determining the dynamics as well as the overall response of the whole network to changes in synaptic strength. For networks whose average synaptic weights for outgoing links have a long-tailed distribution, bursting is observed and the average firing rate of the whole network increases upon inhibition suppression or decreases upon inhibition enhancement. For networks whose average synaptic weights for outgoing links are approximately normally distributed, bursting is not found and the average firing rate of the whole network remains approximately constant upon changes in inhibitory synaptic strength.

## 2. Materials and Methods

We performed numerical simulations of networks of neurons connected by conductance-based synapses (Tomov et al., 2014, 2016; Pena et al., 2016) using networks reconstructed from multi-electrode array recordings taken in a cortical neuronal culture^1^ and their modifications.

*Neuron Model*. Each model consists of *N* neurons. To model the dynamics of a neuron, we used the spiking neuron model proposed by Izhikevich (2003) with the addition of a stochastic noise to mimic external influences. Each neuron, labeled by an index *i*, *i* = 1, 2, …, *N*, is described by two variables: the membrane potential *v*_*i*_ in mV and the membrane recovery variable *u*_*i*_. where *u*_*i*_ accounts for the activation and inactivation of potassium and sodium ions and provides negative feedback to *v*_*i*_. The dynamics of the two variables are governed by two coupled non-linear differential equations


(1) $$\begin{array}{l} \frac{dv_{i}}{dt}=0.04v_{i}^{2}+5v_{i}+140-u_{i}+I_{i}+\alpha \xi \end{array}$$

(2) $$\begin{array}{l} \frac{du_{i}}{dt}=a\left(bv_{i}-u_{i}\right) \end{array}$$

where *t* is time in ms, *I*_*i*_(*t*) is the synaptic current from all pre-synaptic neurons of neuron *i*, ξ is a Gaussian white noise with zero mean and unit variance: 〈ξ(*t*)〉 = 0 and 〈ξ(*t*_1_)ξ(*t*_2_)〉 = δ(*t*_1_ − *t*_2_) and α is the intensity of the noise term. Every time when *v*_*i*_ ≥ 30, neuron *i* fires and sends out a spike, then both variables are reset


(3) $$\{\begin{array}{l} v_{i}\to c\\ u_{i}\to u_{i}+d \end{array}$$

With appropriate values of the four parameters *a*, *b*, *c*, and *d*, the spiking neuron model without the noise term has been shown (Izhikevich, 2007) to be capable to mimic the rich firing patterns exhibited by real neurons from different electrophysiological classes (Nowak et al., 2003; Contreras, 2004). In this study, we focussed on two types of neurons: excitatory regular spiking neurons (*a* = 0.02 and *d* = 8) and inhibitory fast spiking neurons (*a* = 0.1 and *d* = 2), and both types have *b* = 0.2 and *c* = −65 mV (Izhikevich, 2003).

*Synapses*. Neurons in the network are connected by conductance-based synapses such that the current *I*_*i*_(*t*) is given by


(4) $$\begin{array}{l} I_{i}\left(t\right)=G_{i}^{\text{exc}}\left(t\right)\left(V_{E}-v_{i}\left(t\right)\right)+G_{i}^{\text{inh}}\left(t\right)\left(V_{I}-v_{i}\left(t\right)\right) \end{array}$$

where $G_{i}^{\text{exc}}$ and $G_{i}^{\text{inh}}$ are the excitatory and inhibitory conductances, respectively, and *V*_*E*_ = 0 and *V*_*I*_ = −80, both in mV, are the reversal potentials of the excitatory and inhibitory synapses, respectively (Cavallari et al., 2014; Tomov et al., 2014, 2016; Pena et al., 2016). Whenever a pre-synaptic excitatory or inhibitory neuron *j* fires, $G_{i}^{\text{exc}}$ or $G_{i}^{\text{inh}}$ increases by an amount corresponding to the synaptic weight, otherwise it decays with a time constant τ_exc_ or τ_inh_ (Tomov et al., 2014, 2016; Pena et al., 2016):


(5) $$\begin{array}{l} \frac{dG_{i}^{\text{exc}}}{dt}=-\frac{G_{i}^{\text{exc}}}{\tau_{\text{exc}}}+\underset{j,w_{ij}>0}{\sum }w_{ij}\underset{k}{\sum }\delta \left(t-t_{j,k}\right) \end{array}$$

(6) $$\begin{array}{l} \frac{dG_{i}^{\text{inh}}}{dt}=-\frac{G_{i}^{\text{inh}}}{\tau_{\text{inh}}}+\underset{j,w_{ij}<0}{\sum }|w_{ij}|\underset{k}{\sum }\delta \left(t-t_{j,k}\right) \end{array}$$

where τ_exc_ = 5 and τ_inh_ = 6 in ms, *w*_*ij*_ is the synaptic weight of the link from neuron *j* to neuron *i*, with *w*_*ij*_ > 0 for excitatory synapses and *w*_*ij*_ < 0 for inhibitory synapses, and *t*_*j,k*_ is the time of the *k*th spike of pre-synaptic neuron *j*. Solving Equations (5) and (6), we obtain


(7) $$\begin{array}{l} G_{i}^{\text{exc}}=\underset{j,w_{ij}>0}{\sum }w_{ij}\underset{k}{\sum }e^{-\left(t-t_{j,k}\right)/\tau_{\text{exc}}}\theta \left(t-t_{j,k}\right) \end{array}$$

(8) $$\begin{array}{l} G_{i}^{\text{inh}}=\underset{j,w_{ij}<0}{\sum }|w_{ij}|\underset{k}{\sum }e^{-\left(t-t_{j,k}\right)/\tau_{\text{inh}}}\theta \left(t-t_{j,k}\right) \end{array}$$

where the Heaviside step function θ(*t*−*t*_0_) is equal to 1 when *t* > *t*_0_ and zero otherwise. The stochastic differential Equations (1) and (2) together with Equations (4), (7), and (8) were integrated using Euler-Maruyama method (Higham, 2001) with a time step of *dt* = 0.125 ms for a total time of 7, 500 ms. We set the initial values of *v*_*i*_ to be *c* = −65 mV and there is no firing activity when the noise term in Equation (1) is turned off by setting α = 0. The average firing rate of the neurons increases when the noise intensity α increases and we set α = 3 so that the average firing rate is comparable to that measured directly from the multi-electrode array measurements of neuronal culture (see below). We studied the spontaneous activity triggered by the noise. Simulations with smaller *dt* = 0.005 ms and longer total time have been done to check the validity of our simulation results.

*Networks*. We studied six different networks, labeled by I to VI, each of which has *N* = 4095 neurons. Networks I, II, and III were adopted from an earlier study (see text footnote 1) in which the directed effective connectivity of a cortical neuronal culture of rat embryos at different days *in vitro* (DIV) were estimated from voltage measurements recorded by a high density multi-electrode array (HD-MEA). The HD MEA probe (HD-MEA Arena, 3Brain AG) has 4096 electrodes, which are arranged in a 64 by 64 square grid. Spontaneous neuronal activities were recorded for 5 min with the recording device (BioCAM, 3Brain AG) and the associate software (BrainWave 2.0, 3Brain AG) at 7.06 kHz. One electrode was used for calibration purpose so there were 4095 electrodes that recorded 4095 time series of voltage signals. The voltage measurements from the 4095 working electrodes, after noise reduction, were taken as the activities *x*_*i*_(*t*), *i* = 1, 2, …, 4095, of the nodes of a network. Then the connectivity matrix was reconstructed using quantities calculated from *x*_*i*_(*t*)'s using a method developed for reconstructing networks from dynamics (Ching and Tam, 2017) (see Appendix for details). The connectivity matrix elements *w*_*ij*_ of networks I to III are twice of the reconstructed neuronal networks using MEA recordings taken at 25, 45 and 66 DIV (denoted by DIV25, DIV45 and DIV66) respectively. The factor of 2 is used to allow us to get sufficient amount of spiking activity in the relatively short time span of 7,500 ms.

**Figure 1:**
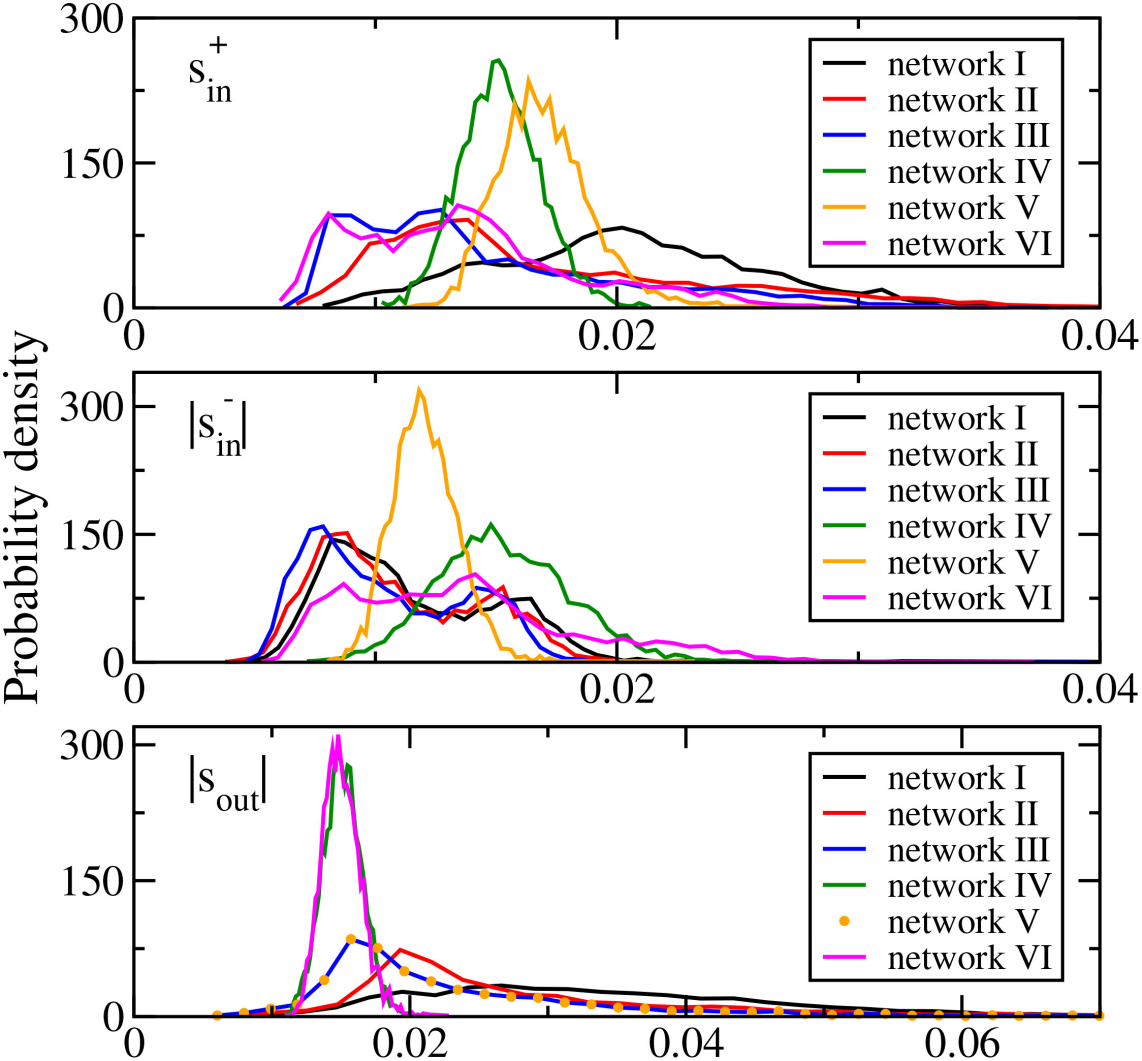
Distributions of average synaptic weights $s_{\text{in}}^{+}$ (upper panel), $\left|s_{\text{in}}^{-}\right|$ (middle panel), and |*s*_out_| (bottom panel) for the six networks studied. In the bottom panel, the distribution of |*s*_out_| of network VI is identical to that of network III and is thus denoted by symbols.

The connection probability of networks I, II, and III is 1.4, 1.1, and 1.5%, respectively. Neurons are either excitatory or inhibitory except for some which have no outgoing links as none was detected in the reconstruction. The fraction of inhibitory neurons is 0.14, 0.21, and 0.28, respectively, for networks I to III and these values are comparable to measured values of 0.15–0.30 in various cortical regions in monkey (Hendry et al., 1987). For each neuron, we define three averages of synaptic weights, the average synaptic weight of excitatory incoming links $s_{\text{in}}^{+}$, the average synaptic weight of inhibitory incoming links $s_{\text{in}}^{-}$ and the average synaptic weight of the outgoing links *s*_out_, by


(9) $$\begin{array}{l} s_{\text{in}}^{+}\left(i\right)=\frac{\sum_{j,w_{ij}>0}w_{ij}}{k_{\text{in}}^{+}\left(i\right)} \end{array}$$

(10) $$\begin{array}{l} s_{\text{in}}^{-}\left(i\right)=\frac{\sum_{j,w_{ij}<0}w_{ij}}{k_{\text{in}}^{-}\left(i\right)} \end{array}$$

(11) $$\begin{array}{l} s_{\text{out}}\left(i\right)=\frac{\sum_{j}w_{ji}}{k_{\text{out}}\left(i\right)}, \end{array}$$

where the excitatory and inhibitory incoming degrees, $k_{\text{in}}^{+}$ and $k_{\text{in}}^{-}$, of a neuron are, respectively, the number of its incoming links of excitatory or positive synaptic weights and inhibitory or negative synaptic weights, and the outgoing degree *k*_out_ of a neuron is the number of its outgoing links. The distributions of the average synaptic weights $s_{\text{in}}^{+}$, $\left|s_{\text{in}}^{-}\right|$, and |*s*_out_| of networks I, II, and III (Figure 1) are skewed and long-tailed, which are generally in accord with the literature (Buzsaki and Mizuseki, 2014). These results show that networks I to III have biologically realistic features.

Networks IV, V, and VI are modifications of network III to allow us to study the possible effects of network topology and the average synaptic weights distributions. Network IV is a random network of the same connection probability as that of network III with its synaptic weights taken from a Gaussian distribution of the same mean and standard deviation as those of network III. Then there is an additional sign adjustment to set the sign of the synaptic weights of all the outgoing links of each neuron to be the same and as in network III. The distributions of the average synaptic weights $s_{\text{in}}^{+}$, $\left|s_{\text{in}}^{-}\right|$, and |*s*_out_| of network IV are all approximately Gaussian (Figure 1). Network V is designed to have the same distribution of |*s*_out_| as network III but with different distributions of $s_{\text{in}}^{+}$ and $s_{\text{in}}^{-}$. It is obtained from network III with the elements of the connectivity matrix in each column replaced by a random permutation of these elements. The same-sign adjustment is also enforced. Such a permutation randomly assigns the outgoing connections of each neuron among all the neurons thus keeping the outgoing degree and |*s*_out_| of each neuron intact. As a result, the distribution of |*s*_out_| of network V is identical to that of network III (see bottom panel of Figure 1). The shuffling randomizes both the number and the synaptic weights of the incoming links of the neurons rendering the distributions of $s_{\text{in}}^{+}$ and $\left|s_{\text{in}}^{-}\right|$ to be approximately Gaussian (see upper and middle panels of Figure 1). Network VI is designed to have similar distributions of $s_{\text{in}}^{+}$ and $s_{\text{in}}^{-}$ as network III but with different distribution of |*s*_out_|. It is obtained from network III in a similar fashion, with the elements of the connectivity matrix in each row replaced by a random permutation and with the same-sign adjustment of the synaptic weights. Similarly, the distribution of |*s*_out_| becomes approximately Gaussian but in this case, the sign adjustment modifies $s_{\text{in}}^{+}$ and $\left|s_{\text{in}}^{-}\right|$ such that their distributions are close to but not the same as those of network III (Figure 1). Table 1 summarizes the features of the three average synaptic weights distributions of the six networks studied.

**Table 1:** A summary of the features of the average synaptic weights distributions of networks I–VI.

	**I**	**II**	**III**	**IV**	**V**	**VI**
long-tailed distribution of $s_{\text{in}}^{+}$	yes	yes	yes	no	no	yes
long-tailed distribution of $\left|s_{\text{in}}^{-}\right|$	yes	yes	yes	no	no	yes
long-tailed distribution of |*s*_out_|	yes	yes	yes	no	yes	no

*Suppression of inhibition*. For each network, we calculated the standard deviation of all the inhibitory synaptic weights with *w*_*ij*_ < 0 and denoted the result as σ. We applied three levels of uniform suppression of inhibition by replacing every negative *w*_*ij*_ by *w*_*ij*_ + *kσ* for (i) *k* = 0.25, (ii) *k* = 0.5, and (iii) *k* = 1. When the magnitudes of the inhibitory synaptic weights are decreased, an inhibitory neuron would not be turned into an excitatory neuron thus we enforced an additional condition: if *w*_*ij*_ + *kσ* > 0, then that negative *w*_*ij*_ is replaced by zero. We carried out simulations for each of the networks as well as the three levels of inhibition suppression and recorded the number of spikes and the times at which the spikes occur for all the 4095 neurons in every simulation.

*Calculation of firing rates from MEA recordings*. By applying the Precise Timing Spike Detection algorithm (Maccione et al., 2009) in the BrainWave software associated with the recording device of the MEA probe, we detected spikes in the MEA recordings DIV25, DIV45, and DIV66 and calculated the firing rates of the measurements of each of the 4095 electrodes. The noise intensity α in Equation (1) is set to be 3 for the simulations so that the average firing rates of the whole network for networks I–III are comparable to the array-wide average firing rates calculated from the MEA recordings.

Three additional sets of MEA recordings were taken, respectively, after 5 μM, 15 μM, and 30 μM of bicuculline were added to the neuronal culture at 66 DIV. We calculated the firing rates of these three sets of MEA recordings. Our simulation results will be useful for understanding the effects of bicuculline on firing rates of neuronal networks.

## 3. Results

**Figure 2:**
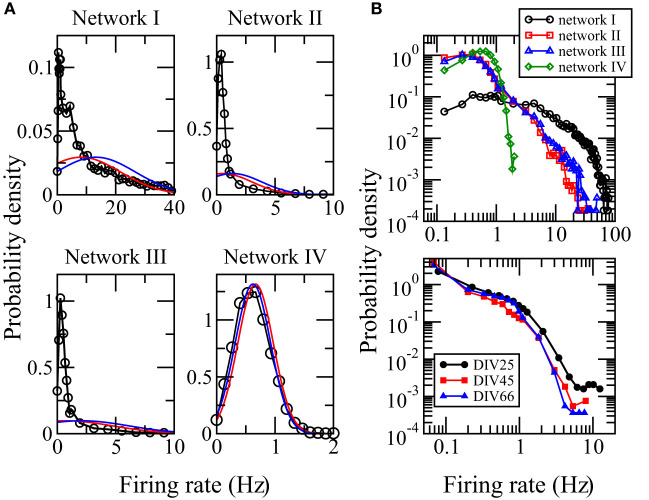
Distributions of firing rates. **(A)** Distributions calculated from the simulation data for networks I–IV. The red curve is a Gaussian distribution with the same median and standard deviation while the blue curve is a Gaussian distribution with the same mean and standard deviation. **(B)** Comparison of the distributions of firing rates for networks I–IV (upper panel) with those calculated using the spikes detected in the measured MEA recordings DIV25, DIV45, and DIV66 (bottom panel) from which networks I–III were reconstructed. The data points for zero firing rate are removed. A good resemblance of the long tails in the distributions for networks I–III can be seen.

The activity of a neuron is often measured by its firing rate, which is defined as the total number of spikes recorded in a certain time interval *T* divided by *T*. We used the whole computational time interval *T* = 7, 500 ms to calculate the firing rates. The distribution of firing rates of neurons in local cortical networks has been reported to be skewed with long tails (Shafia et al., 2007; O'Connor et al., 2010; Peyrache et al., 2012; Buzsaki and Mizuseki, 2014). We first study whether networks I, II, and III, adopted from networks reconstructed from the MEA recordings of a neuronal culture, can reproduce these features in the spiking neuron model. Figure 2A shows the distributions of firing rate for networks I–III. They are highly skewed, long tailed, and clearly deviate from Gaussian distributions with the same mean or median and same standard deviation. These features indicate that the spiking activities of the whole network are dominated by a small fraction of neurons. The distribution of firing rate depends crucially on the distribution of synaptic weights of the network (Roxin et al., 2011). For the random network IV whose average synaptic weights obey an approximately Gaussian distribution, Figure 2A shows that its distribution of firing rates is neither skewed nor long-tailed but is well approximated by a Gaussian distribution with the same mean and standard deviation. We show the distributions of firing rates for networks I–IV in a log-log plot in Figure 2B. The good resemblance of the long tails in the distributions for networks I–III with those calculated directly from the MEA recordings (Figure 2B) further supports that these three networks have biologically realistic features.

**Figure 3:**
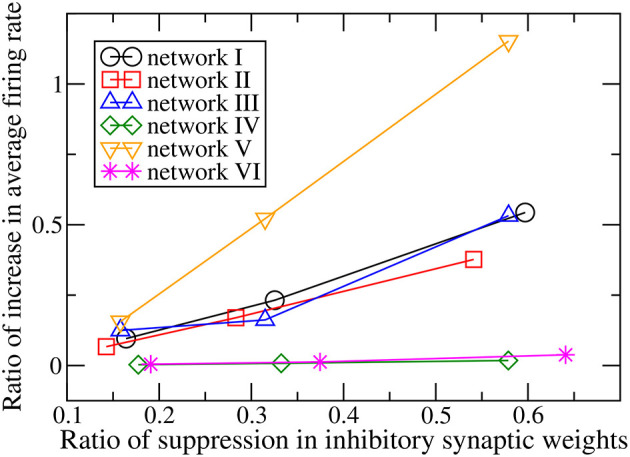
Dependence of the ratio of the increase of the average firing rate of the whole network on the ratio of suppression in inhibitory synaptic weights for the six networks.

**Figure 4:**
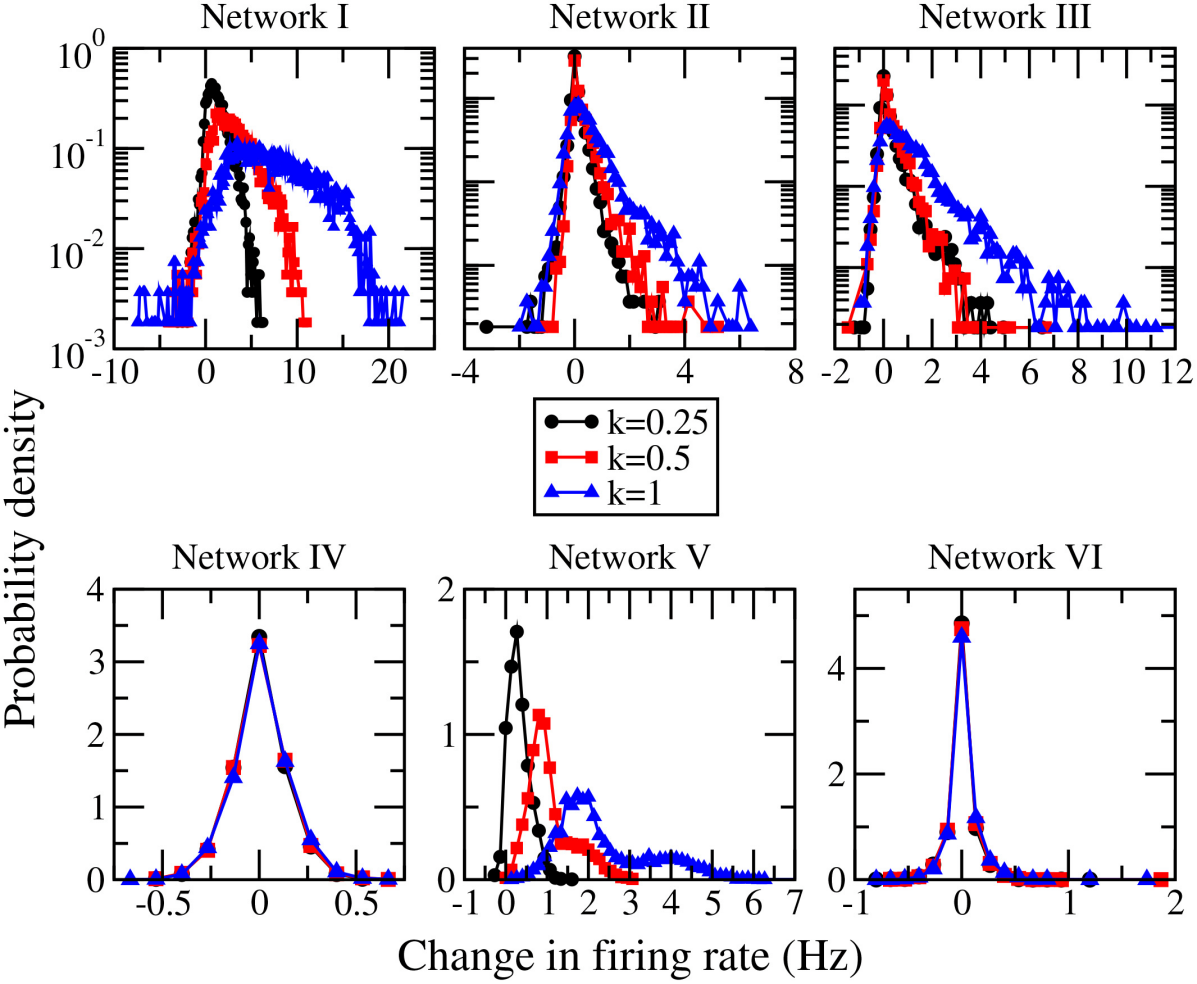
Distributions of the change in firing rate for the three different levels of suppression of inhibition for networks I–VI.

**Figure 5:**
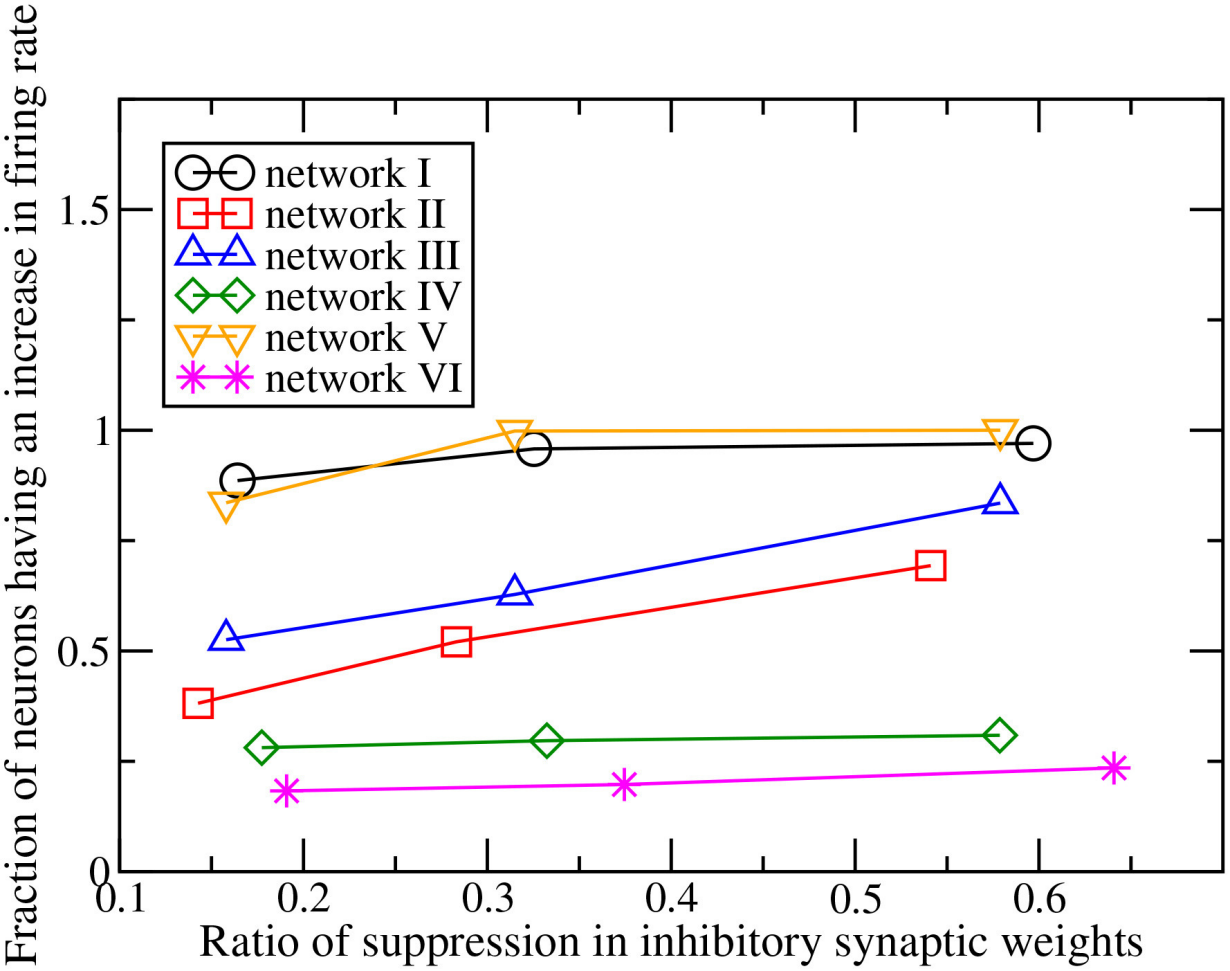
Dependence of the fraction of neurons having an increase in firing rate on the ratio of suppression in inhibitory synaptic weights for the six networks.

When the magnitudes of all the inhibitory synaptic weights are decreased, the magnitudes of the presynaptic inhibitory synapses of every neuron are decreased. Thus one would naturally expect the firing rate of every neuron to be enhanced and that the average firing rate of the whole network to increase with the level of inhibition suppression. Figure 3 shows the dependence of the average firing rate of the network on the ratio of suppression in inhibitory weights for all the networks. The ratio of suppression in inhibitory weights is equal to the ratio of the decrease in the average magnitude of all the inhibitory weights to the average magnitude of all the inhibitory synapses when there is no suppression. The average firing rate increases for most of the networks as expected but surprisingly, it remains approximately unchanged for networks IV and VI. Inspection of the change in firing rates of the individual neurons within a network reveals that the responses of neurons are heterogeneous: while the firing rates of some neurons are enhanced as expected, the firing rates of the other neurons decrease or do not change. Such heterogeneous responses are found in all the six networks. Figure 4 shows the distributions of change in firing rate at the three applied levels of suppression. It can be seen that there exists a non-zero fraction of neurons whose firing rate decreases for all the networks at the lowest applied level of suppression (*k* = 0.25). For network V, the firing rates of all neurons increase or do not change at the two highest applied levels of suppression (*k* = 0.5 and *k* = 1). For the average firing rate of the whole network to have a net increase, the distribution of the change in firing rate has to be asymmetric and skewed toward positive changes in firing rate. This is indeed the case for all except networks IV and VI. For networks IV and VI, the distribution of the change in firing rate is symmetric about zero and there is thus no net change in the average firing rate or the overall spiking activity in these two networks. Moreover, the distributions of the change in firing rate have a very weak dependence on *k* and the fraction of neurons having an increase in firing rate is approximately constant as *k* increases (Figure 5). For networks I–III and network V, most of the neurons exhibit an increase in their firing rates and the fraction of neurons having an increase in firing rate increases with the ratio of suppression in inhibitory synaptic weights (Figure 5).

What determines the different responses of the individual neurons within a network? A first guess might be the group of neurons with an increase in firing rates and the group of neurons with a decrease in firing rates differ in their network features such as degree and average synaptic weights. However, this possibility has to be ruled out since heterogeneous responses are found in all the networks including network V, which is a random network, and the nodes in a random network have similar in- and out-degrees and average synaptic weights. We calculated the distributions of the incoming and outgoing degrees and the average synaptic weights of incoming and outgoing links separately for the two groups of neurons, one with an increase in firing rate and the other with a decrease, in networks I–III and indeed found no significant differences. Moreover, heterogeneous responses are found among excitatory neurons and among inhibitory neurons and no correlation is found between the sign of the change in firing rate and the nature of the neuron.

**Figure 6:**
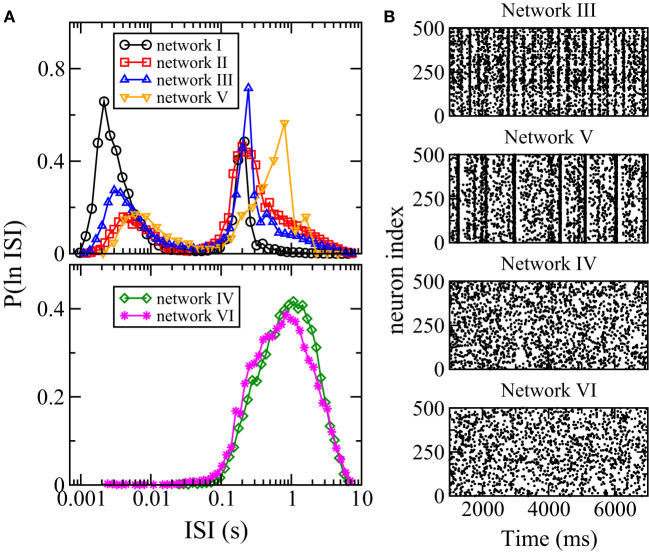
Distribution of interspike intervals (ISI) and bursting. **(A)** Distribution P(ln ISI) for the six networks. **(B)** Raster plots of 500 randomly chosen neurons in networks III–VI. When distribution of ISI is bimodal, bursting is observed.

Besides firing rate, another measure of spiking activity is the inter-spike interval (ISI). To study neuronal variability, one common method is to study the distribution of ISI or ln(ISI), the logarithm of ISI. In particular, multi-scale bursting activities of a neuronal network can be revealed by a bimodal ln(ISI) distribution with one peak at shorter ISI for spikes within each burst and another peak at longer ISI for spikes between consecutive bursts (Cocatre-Zilgien and Delcomyn, 1992; Selinger et al., 2007). Figure 6A shows the distributions of ln(ISI) for all the six networks. For networks I, II, III, and V, the distributions are bimodal with one peak at ISI of the order of ms and another peak at larger ISI of order of 0.1 s, thus these networks have bursting activities as can be seen directly in the raster plots (Figure 6B). The ln(ISI) distributions of networks IV and VI are unimodal (Figure 6A) and these two networks have no bursts (Figure 6B). Hence, there is an interesting correlation between the bursting dynamics of a network and its overall response to changes in synaptic weights: the average firing rate of the whole network has a net change for networks with bursting but remains unchanged for networks without bursts when the inhibitory synaptic weights are varied.

The network architecture and the synaptic weights distribution are expected to affect the dynamics of the network and its response to changes in synaptic weights but it is not obvious which specific network feature plays a crucial role. By comparing the features of the distribution of the average synaptic weights of the networks as summarized in Table 1, we can conclude that the distribution of average synaptic weights of the outgoing links *s*_out_ of a network is crucial in determining the dynamics as well as the overall response of the whole network to changes in synaptic strength. For networks whose *s*_out_'s have a long-tailed distribution, bursting is observed and the average firing rate of the whole network increases upon inhibition suppression or decreases upon inhibition enhancement. For networks whose *s*_out_'s are approximately normally distributed, bursting is not found and the average firing rate of the whole network remains approximately constant upon changes in inhibitory synaptic strength.

**Figure 7:**
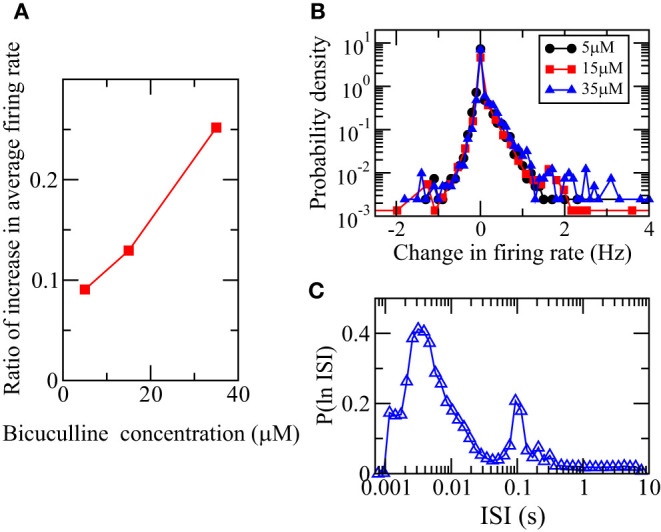
Heterogeneous responses of spiking activity of neuronal culture at 66 DIV to the addition of bicuculline. **(A)** Dependence of the ratio of the increase of the average firing rate of all 4,095 MEA channels for 66 DIV on the concentration of bicuculline. **(B)** Distributions of the change in firing rate of all the channels for the three different concentrations. **(C)** Distribution of logarithm of ISI, P(ln ISI), calculated using the spikes detected from the MEA recording DIV66.

Using the calculated firing rates from the MEA recordings of the 4,095 electrodes taken at 66 DIV after three different concentrations of bicuculline were added, we found that the array-wide average firing rate increases with the addition of bicuculline as previously reported (Eisenman et al., 2015). Figure 7A shows that the array-wide average firing rate increases as the bicuculline concentration increases. Bicuculline is a competitive GABA_A_ receptor antagonist that blocks the inhibitory action of the neurotransmitter GABA (Johnston, 1996). The blocking action of bicuculline on the receptors of GABA can be crudely modeled by a suppression of inhibitory synaptic weights and our simulation results thus suggest that the responses in firing rate would be heterogeneous. Bicuculline has indeed been reported to exhibit a heterogeneous effect on firing rate in rat hippocampal neuronal networks (Sokal et al., 2000). We calculated the distributions of change in firing rates of the recordings of individual electrodes. Figure 7B shows that there are both positive and negative changes and confirms that bicuculline exhibits a heterogeneous effect on firing rate. Since there is a net change in the array-wide average firing rate on the bicuculline concentration, based on our simulation results, we would expect that the ln(ISI) distribution calculated from the MEA recording DIV66 should be bimodal. Figure 7C confirms this prediction.

## 4. Discussion

One of the most fascinating properties of the brain is neuroplasticity, its ability to change the synaptic strength of connections and/or to form new connections in its neuronal circuits in response to experience and stimuli. Specifically, synaptic plasticity, the activity-dependent modification of the synaptic strength of connections, has been proposed to play a central role in learning and memory over the past century. Changes in synaptic strength are also thought to be crucial during early development of the brain. Many forms and mechanisms of synaptic plasticity have been described in which synaptic strength can be either enhanced or depressed and these changes can be either short term or more long lasting (Brown et al., 1990; Bear and Malenka, 1994; Malenka and Nicoll, 1999; Bi and Poo, 2001; Bi, 2002; Zucker and Regehr, 2002; Citri and Malenka, 2008; Bailey et al., 2015). It is natural to expect that an enhancement of synaptic strength would lead to an enhancement in activity and a depression of synaptic strength would lead to a depression in activity and thus activity-dependent synaptic plasticity alone would lead to instability (Abbott and Nelson, 2000) and a number of stabilization mechanisms have been suggested (Chen et al., 2013; Bannon et al., 2020). There are, however, experimental and numerical results suggesting that changes in the strength of the synapses onto a neuron do not always lead to changes in the spiking activity of the neuron (Prinz et al., 2003). Moreover, the response of a neuron to changes in the synaptic strength is likely to be influenced by its interactions with other neurons in a network. For a full understanding of synaptic plasticity, it is thus useful to study how the effects of changes in synaptic strength on the dynamics of neurons in neuronal networks.

In this work, we carried out numerical simulations of networks of thousands of spiking neurons with conductance-based synapses and showed that a uniform suppression of the inhibitory synaptic weights does not lead to an increase in the firing rate of all the neurons within a network. In comparison with networks with an applied suppression of inhibition, the original networks could be viewed as networks with an applied enhancement of inhibition. Thus our results imply that heterogeneous responses do not only occur for a suppression of inhibition but also for an enhancement of inhibition. That is, neurons in a network respond differently to changes in inhibitory synaptic weights. As a result, a suppression or an enhancement of the magnitudes of the synaptic weights of all presynaptic inhibitory synapses of a neuron in a network does not always lead to an increase or decrease in the firing rate of this neuron. Hence, activity-dependent modification of synaptic strength does not necessarily generate a positive feedback on the dynamics of neurons connected in a network and thus synaptic plasticity does not necessarily lead to unstable runaway synaptic dynamics in neuronal networks.

The effects of different drugs on the spiking and bursting activities of neuronal networks have been commonly studied (e.g., Sokal et al., 2000; Eisenman et al., 2015). It has been found that bicuculline, a drug that blocks the receptors of inhibitory neurotransmitter GABA, has a heterogeneous effect on firing rate in a rat hippocampal neuronal network (Sokal et al., 2000). Our simulations results showing a heterogeneity in the responses to a suppression in inhibition thus suggests a heterogeneity in the action of bicuculine when its blocking action of the GABA receptors is crudely modeled as a decrease in magnitudes of all inhibitory synaptic weights. Our study can be further used to understand the effects of bicuculline on bursting.

Such heterogeneous responses are found in all networks studied including a generic random network (network V) in which all the nodes have similar degrees and average synaptic weights described by an approximately Gaussian distribution with small standard deviation (see Figure 1). Moreover, heterogeneous responses are found among excitatory neurons and also among inhibitory neurons. We have indeed found that the two groups of neurons that have opposite changes in the firing rate upon changes in inhibitory synaptic strength in networks I–III have similar distributions of degrees and average synaptic weights. These results thus indicate that whether the firing rate of a neuron increases or decreases upon a changes in inhibition is not a simple consequence of its network features or whether it is excitatory or inhibitory.

The firing rate of neuron *i* is controlled by the synaptic current *I*_*i*_(*t*), which depends on the excitatory and inhibitory conductances. The inhibitory conductance $G_{i}^{\text{inh}}$ depends not only on the magnitude of the synaptic weights |*w*_*ij*_| but also on the firing history of the presynaptic inhibitory neurons of neuron *i*. Thus $G_{i}^{\text{inh}}$ could increase even when the magnitudes of all the synaptic weights of the presynaptic inhibitory synapses are decreased if some of the presynaptic inhibitory neurons fire more frequently. Similarly, the excitatory conductance $G_{i}^{\text{exc}}$ could decrease even when the magnitudes of all the synaptic weights of the presynaptic excitatory synapses are fixed if the firing rate of some of the presynaptic excitatory neurons decreases. This explains why *I*_*i*_(*t*) can decrease and leads to a decrease in the firing rate of neuron *i* even when all the synaptic weights of its presynaptic inhibitory synapses are suppressed. The response of an individual neuron to changes in synaptic strength is affected by the firing activity of its presynaptic neurons, which is in turn affected by the firing activity of their own presynaptic neurons, and hence the heterogeneous responses are the result of the interactions among neurons in the network.

Detailed exploration of how the architecture of a neuronal network, which describes the inter-actions among neurons, gives rise to the heterogeneous responses should provide new insights on synaptic plasticity. For such an exploration to be fruitful, it is important to use neuronal networks with biologically realistic connections rather than generic networks like random networks that are often used in computational studies. Our simulations show that unrealistic dynamics are obtained for a random network (network V) in that there is no bursting and no change in the overall average firing rate upon variations of the inhibitory synaptic strength.

In addition to the heterogeneity in the responses of individual neurons within a network, our results show that network structure induces a variability in the overall response of the whole network to changes in inhibitory synaptic strength. It is not surprising that there is a relationship between network structure and dynamics but it is challenging to pin down which specific network feature plays a crucial role. Using biologically realistic networks and their modifications enables us to address this challenge. The reconstructed networks from MEA recordings of neuronal culture (networks I–III) have biologically realistic long-tailed distributions of average synaptic weights. By comparing the dynamics of these networks with those of the modified ones (networks IV–VI), we are able to conclude that a long-tail distribution of average synaptic weights of the outgoing links *s*_out_ is the crucial feature that gives rise to bursting in neuronal networks and determines the overall response of the whole network to changes in synaptic strength. Understanding how a long-tailed distribution of *s*_out_ gives rise to bursting and why networks lacking such a feature would experience little or no change in the overall average firing rate upon changes in synaptic strength are interesting problems to be explored in future studies.

## Data Availability Statement

The raw data supporting the conclusions of this article will be made available by the authors, without undue reservation.

## Code Availability Statement

The numerical code written in Python can be found in GitHub: https://github.com/escching/NetworkSpikingModel.

## Author Contributions

HL and GC: modified the computer code, ran the simulations, and did the data analysis. EC: designed the work, did the data analysis, and wrote the manuscript. All authors contributed to the article and approved the submitted version.

## Funding

Our work was supported by the Hong Kong Research Grants Council under grant no. CUHK 14304017.

## Conflict of Interest

The authors declare that the research was conducted in the absence of any commercial or financial relationships that could be construed as a potential conflict of interest.

## Publisher's Note

All claims expressed in this article are solely those of the authors and do not necessarily represent those of their affiliated organizations, or those of the publisher, the editors and the reviewers. Any product that may be evaluated in this article, or claim that may be made by its manufacturer, is not guaranteed or endorsed by the publisher.

## Footnotes

^1^Sun, C., Lin, K. C., Yeung, C. Y., Huang, Y.-T., Lai, P.-Y., Chan, C., et al. (2021). Revealing directed effective connectivity of cortical neuronal networks from measurements, under review. *Phys. Rev. E*.

## 5. Appendix

The recorded 4,095 time series of voltage signals are denoted as *y*_*i*_(*t*), *i* = 1, 2, …, 4, 095. To reduce the effect of measurement noise, a moving average filter was first applied to obtain *x*_*i*_(*t*) = [*y*_*i*_(*t*) + *y*_*i*_(*t* + Δ)]/2, where Δ = 0.142 ms is the sampling time interval for *i* = 1, 2, …, 4, 095. Then *x*_*i*_(*t*) is treated as the activity of node *i* of a network of *N* = 4, 095 nodes with their connections given by the connectivity matrix elements ${\tilde{w}}_{ij}$. That is, ${\tilde{w}}_{ij}\neq 0$ is the weight of the connection from node *j* to node *i* and ${\tilde{w}}_{ij}=0$ when there is no connection from node *j* to node *i*. We assume no self-connections such that ${\tilde{w}}_{ii}=0$.

Using *x*_*i*_(*t*)'s, we calculated two matrices, the time-lagged covariance matrix **K**(τ) whose elements are given by

(12)
$$\begin{array}{l} K_{ij}\left(\tau \right)=\langle \left[x_{i}\left(t+\tau \right)-\langle x_{i}\left(t+\tau \right)\rangle \right]\left[x_{j}\left(t\right)-\langle x_{j}\left(t\right)\rangle \right]\rangle \; \end{array}$$

using τ = Δ and the equal-time covariance matrix **K**(0), whose elements are given by Equation (12) with τ = 0. Here, 〈…〉 denotes a time average. Then, we calculated

(13)
$$\begin{array}{l} \text{M}=\frac{1}{\tau }\log\left(\text{K}\left(\tau \right)\text{K}{\left(0\right)}^{-1}\right) \end{array}$$

where log is the principal matrix logarithm.

For systems whose dynamics are described by a set of coupled stochastic differential equations and approach a fixed point in the noise-free limit, it has been derived that (Ching and Tam, 2017)

(14)
$$\begin{array}{l} {\tilde{w}}_{ij}\approx M_{ij}\;i\neq j \end{array}$$

Relation (Equation (14)) thus implies that for each node *j*, the off-diagonal elements *M*_*ij*_, with *i* ≠ *j*, would separate into two groups corresponding to ${\tilde{w}}_{ij}=0$ (no links from node *j* to node *j*) and ${\tilde{w}}_{ij}\neq 0$ (links from node *j* to node *i* with weights ${\tilde{w}}_{ij}$). Motivated by this result, a method has been developed to estimate ${\tilde{w}}_{ij}$ by performing clustering analysis of *M*_*ij*_ into two groups for each node *j*. This covariance-relation based method of recovering the connectivity matrix has been validated by numerical simulations not only for systems that have a fixed point in the noise free limit but also for some systems that do not approach a fixed point in the noise-free limit, including especially systems that obey the FitzHugh-Nagumo dynamics that is commonly used to model neurons (Ching and Tam, 2017; Tam, 2017).

This method was adopted (see text footnote 1) to estimate ${\tilde{w}}_{ij}$ from MEA recordings taken at different Days in Vitro (DIV). In each case, the distribution of *M*_*ij*_ with *i* ≠ *j* for each node *j* is fitted by a sum of two Gaussian component distributions using MATLAB “fitgmdist” and the Gaussian component of the larger proportion or the Gaussian component with the average closer to zero is taken as the unconnected component of ${\tilde{w}}_{ij}=0$. The probability *p*_*i*_ of each of the *M*_*ij*_ values belonging to the unconnected component is then obtained by using MATLAB “cluster.” If *p*_*i*_ > 0.5, then ${\tilde{w}}_{ij}=0$ and there is no link from node *j* to node *i*. Otherwise if *p*_*i*_ ≤ 0.5, then there is a link from node *j* to node *i* with ${\tilde{w}}_{ij}=M_{ij}-{\langle M_{kj}|{\tilde{w}}_{kj}=0\rangle }_{k}$ where ${\langle M_{kj}|{\tilde{w}}_{kj}=0\rangle }_{k}$ is the average over *k* of those *M*_*kj*_ values that are estimated to correspond to ${\tilde{w}}_{kj}=0$. This procedure is repeated for all the nodes *j* to estimate all the off-diagonal elements ${\tilde{w}}_{ij}$ with *i* ≠ *j*. In the event that, the two Gaussian component distributions are not well separated, the distribution of *M*_*ij*_ is fitted again by one single Gaussian distribution with the outliers inferred as the connected component with ${\tilde{w}}_{ij}\neq 0$.

The *w*_*ij*_'s of networks I, II, and III are twice of ${\tilde{w}}_{ij}$ reconstructed from MEA recordings DIV25, DIV45, and DIV66, respectively.
